# Tactile stimulation in the delivery room: past, present, future. A systematic review

**DOI:** 10.1038/s41390-022-01945-9

**Published:** 2022-02-05

**Authors:** M. Kaufmann, L. Mense, L. Springer, J. Dekker

**Affiliations:** 1 Division of Neonatology and Paediatric Intensive Care Medicine, Department of Paediatrics, Medical Faculty, TU Dresden, Dresden, Germany; 2 Saxony Center for Feto-Neonatal Health, Medical Faculty, TU Dresden, Dresden, Germany; 3 Division of Neonatology, Department of Paediatrics, University Children’s Hospital Tuebingen, Tuebingen, Germany; 4 Division of Neonatology, Department of Paediatrics, Leiden University Medical Center, Leiden, The Netherlands

## Abstract

In current resuscitation guidelines, tactile stimulation is recommended for infants with insufficient respiratory efforts after birth. No recommendations are made regarding duration, onset, and method of stimulation. Neither is mentioned how tactile stimulation should be applied in relation to the gestational age. The aim was to review the physiological mechanisms of respiratory drive after birth and to identify and structure the current evidence on tactile stimulation during neonatal resuscitation. A systematic review of available data was performed using PubMed, covering the literature up to April 2021. Two independent investigators screened the extracted references and assessed their methodological quality. Six studies were included. Tactile stimulation management, including the onset of stimulation, overall duration, and methods as well as the effect on vital parameters was analyzed and systematically presented. Tactile stimulation varies widely between, as well as within different centers and no consensus exists which stimulation method is most effective. Some evidence shows that repetitive stimulation within the first minutes of resuscitation improves oxygenation. Further studies are warranted to optimize strategies to support spontaneous breathing after birth, assessing the effect of stimulating various body parts respectively within different gestational age groups.

## Introduction

### Historical overview

Approximately 5–10% of the newborns and the majority of premature infants need interventions to assist the neonatal transition and ~1% receive intensive resuscitation during delivery room management.^1–3^ In the past decades, many papers have been published describing practices used in the delivery room to stimulate and support breathing. During these years, stimulation practices have changed significantly.

In the early years of neonatal resuscitation, brutal and astonishing strategies were applied in order to support and provoke neonatal transition, such as tongue pulling, immersion into cold water, shaking, clapping, pinching, milking the trachea, rectal dilatation, and oxygenated air administration into the stomach.^4,5^ In the eighteenth and nineteenth centuries, the discovery of oxygen and the importance of lung ventilation led to a change in practice.^6,7^ Blundell described in 1834 the need for rapid and routine neonatal intubation to stabilize the depressed infant as early as possible, which made the use of physical stimulation to support spontaneous breathing fade into the background.^8^

With a better understanding of neonatal physiology about a century later, the use of methods to stimulate depressed infants gained ground in addition to the use of invasive ventilation. For example, Hess and Lundeen recommended the administration of drugs such as caffeine sodium benzoate and the inhalation of aromatic spirity of ammonia.^7^ Furthermore, the importance of physical stimulation was highlighted by Bloxsom et al., who developed the positive-pressure-oxygen air lock for resuscitation after the cesarian section.^9^ Infants were put into a cyclical steel chamber that was subsequently infused with humidified, heated 60% oxygen, and cycled positive pressure. Bloxsom therewith aimed to imitate uterine contractions as he suggested they have a direct stimulatory effect on the chest wall and lung during vaginal delivery.^9^ More importantly, the need to focus on the physiological transition of infants was highlighted by the implementation of the Apgar score in 1958, which is used routinely to date to clinically evaluate the infant,^10,11^ even though its transferability in preterm infants has been critically discussed.^12–15^

When more knowledge about physiology during the neonatal transition was gained, it became clear that therapies for asphyxiated term infants could not be simply adopted to preterm infants. The need for respiratory support in extremely preterm infants is due to their immaturity of both the musculoskeletal and pulmonary system.^16,17^ Applying invasive mechanical ventilation to preterm infants resulted in irreversible lung injury and worse neurological outcome.^18–20^ This led to reconsiderations of respiratory support in the delivery room. In recent years, the approach has changed to non-invasive breathing support using continuous positive airway pressure for spontaneously breathing infants and positive pressure ventilation (PPV) for infants with inadequate breathing.^21,22^

This change in practice increased the need for other medical and non-medical interventions to stimulate spontaneous breathing. It became clear from animal studies that tactile stimulation (TS) could be beneficial^23^ and that shortly after birth, laryngeal closure seems to impede non-invasive ventilation.^24^ The larynx opens during a spontaneous breath and will only remain predominantly open once a stable spontaneous breathing pattern has been established.^24^ Once again, this highlights the importance of the use of interventions to stimulate spontaneous breathing.

In today’s clinical practice, neonatal resuscitation follows a recommended algorithm published in current guidelines of the American Heart Association^25^ and the European Resuscitation Council.^21^ The International Liaison Committee on Resuscitation regularly issues scientific advice on neonatal resuscitation, adapted to the current state of knowledge regarding feto-neonatal transition.^26^ In these guidelines, the use of TS is mentioned to support spontaneous breathing.^26^ Applying TS repetitively to the back, the chest, or the soles of the feet could be beneficial for spontaneous breathing, but the level of evidence is graded as low.^21,27^ These guidelines recommend future studies to determine appropriate methods of TS. Patterns of active stimulation in preterm infants vary highly and their effectiveness in improving respiratory stability is unclear.^12,28,29^ Furthermore, video analysis of management in the delivery room revealed that manipulations (which subsequently led to a stimulation) are very common. Whereas term newborns remained without any manipulation for 24% (0–69) of the analyzed time,^30^ it was <5% of the time in preterm infants.^31^

With this systematic review, we identify, appraise, and synthesize the available evidence on TS in the delivery room and identify gaps in knowledge on this topic that can be used to guide future studies.

### The onset of breathing in newborns: physiological changes

During feto-neonatal transition, lung aeration and the onset of a stable respiratory pattern are of utmost importance. Fetal breathing movements (FBM) start early in pregnancy and become more common at higher gestational ages. FBM are modified by various factors, such as quiet-active cycles of the fetus, maternal glucose levels, and maternal gas exchange.^32^ Antenatal lung development is stimulated by changes in intrathoracic pressures caused by FBM and glottis closure.^33^

Glottis closure is a common phenomenon in fetuses that controls the efflux of fetal lung fluid into the pharynx.^34^ Postnatally, glottis closure is a double-edged sword: during the undisturbed transition and with stable breathing patterns, the glottis is open during inspiration and most of the expiration. For a short time during expiration, the glottis is closed actively which may increase the intrapulmonary pressures and support transition.^24^ In disturbed transition, periods of apnea are common: Glottis and epiglottis close during apnea and obstruct the airway.^24^ The effectiveness of PPV is severely impaired since the pressures do not overcome the closed glottis and ventilation of the lung has not been observed. Therefore, PPV as a single intervention in apneic newborns might be insufficient and stimulation to initiate spontaneous respiration might be beneficial.

**Fig. 1:**
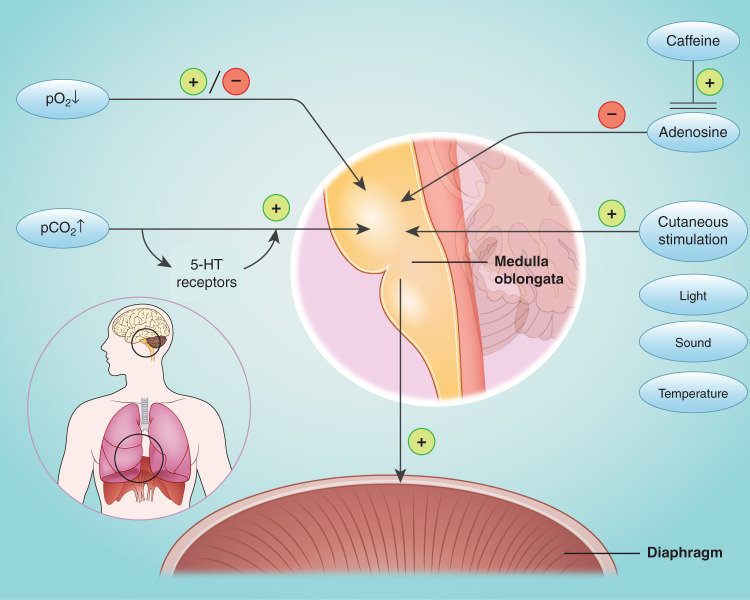
Modifying factors of neonatal respiratory drive.

In animal models and clinical studies, different factors have been described to influence respiratory drive after birth (Fig. 1): although hypoxia is shown to be a respiratory stimulus in mature infants, an impaired response of peripheral chemoreceptors to hypoxia most likely results in inhibition of breathing in preterm infants at birth.^35–37^

Adenosine inhibits spontaneous breathing^38,39^ while caffeine, which is an antagonist at adenosine receptors,^40^ is a potent and regularly used respiratory stimulant. In rodents, mothers stimulate their offspring instinctively^23^ and caregivers’ stimulation effectively improves respiratory drive.^41^ Despite being mentioned in neonatal resuscitation guidelines for many years, postnatal stimulation has been sparsely investigated in humans.

## Methods

### Search strategy

To identify all relevant studies, published until April 2021, a structured search through PubMed was performed without limitations for country or language. Also, animal studies were included. The following search strategy was used: (“Physical Stimulation”[Mesh] OR “physical stimul*”[tw] OR “tactile stimul*”[tw] OR “kinesthetic stimul*”[tw] OR ((physical[ti] OR tactile[ti] OR kinesthetic[ti]) AND stimul*[ti])) AND (“Infant, Newborn”[Mesh] OR “Intensive Care, Neonatal”[Mesh] OR “Intensive Care Units, Neonatal”[Mesh] OR “Neonatal Nursing”[Mesh] OR infant*[tw] OR newborn*[tw] OR neonat*[tw] OR prematur*[tw] OR preterm*[tw] OR ELBW[tiab] OR LBW[tiab]) AND (“at birth”[tw] OR “Delivery Rooms”[Mesh] OR “Delivery Room*”[tw] OR “delivery unit*”[tw] OR “delivery suite*”[tw] OR “delivery chamber*”[tw] OR “delivery bed*”[tw]). All references were entered into EndNote v20.1 (Clarivate, Philadelphia).

### Inclusion and exclusion criteria

#### Patients

We integrated studies of preterm as well as term neonates. Animal studies, in particular, two studies about the stimulation of newborn rats, were excluded as they did not provide relevant information and references on neonatal stimulation in human beings.

#### Intervention

Studies were included that described the management of TS in the delivery room. Studies in which TS was used to stimulate spontaneous breathing performed on the neonatal intensive care unit (NICU) were excluded.

#### Outcome

Studies were included that describe the current TS management in the delivery room and the effects of TS on spontaneous breathing and vital parameters, such as heart rate and oxygen saturation.

#### Type of study

Because of the limited number of studies, we included randomized controlled trials (RCTs), but also observational, retrospective studies.

A three-stage inclusion process was applied. Initially, the references found by the literature search were screened by title and abstract to meet the inclusion criteria by one of the authors (J.D.). In the second stage, a second reviewer (M.K.) examined all full texts of the articles selected in the first stage. In case of doubt, the full texts were screened by a second author (J.D.), and differences were discussed. Finally, all selected references were screened by all the authors to determine if they fulfilled the inclusion criteria and could be used and analyzed in this review.

### Quality assessment

The methodological quality of the included studies was assessed independently by two reviewers (M.K. and J.D.). For RCTs, the Cochrane Risk-of-Bias (RoB) tool 2 was used. An overall quality rating was assigned, ranging from high to low risk of bias.

Due to the designs of the observational, retrospective analyses, the authors decided not to rate them but to assess their risk of bias as high.

### Data analysis and synthesis

Two authors (M.K. and L.S.) extracted data from included references and arranged them systematically in a table (Table 1). Due to their study designs, extracted data could not be pooled in a meta-analysis and only a qualitative synthesis followed.

**Table 1:** Overview of the current evidence on TS in the delivery room.

First author, reference number	Study characteristics	Patient characteristics	Results	
Dekker et al.^29^	Retrospective study 01/2007–06/2016	Stimulation group: GA 29^0/7^ [27^3/7^; 30^2/7^] weeks *n* = 164	Analyzed time frame: first 7 min of resuscitation Details of TS: 67% stimulation rate onset after 114 s [73 s; 182 s] episodes per infant 3 [1; 5] duration per episode 8 s [4 s; 16 s] overall duration 32 s [15 s; 64 s] Methods (%—of overall episodes): 68% foot rub 12% back rub 2% foot flick 9% combination 8% others Physiological and clinical outcomes: 18% heart rate recovery (HR, >100 bpm) and/or increased breathing efforts 7% were intubated*	
Gaertner et al.^28^	Retrospective study 2004–2006	<30 weeks of GA GA 27^0/7^ [25^0/7^; 28^0/7^] weeks *n* = 60 ≥30 weeks of GA GA 34^5/7^ [31^0/7^; 39^0/7^] weeks *n* = 60	Analyzed time frame: first 5 min of resuscitation Details of TS: 63% stimulation rate 58% stimulation within the first minute Methods (%—of overall episodes): 41% drying 37% chest rub 12% back rub 10% foot flick Physiological and clinical outcomes: HR increase (before and 5 s after stimulation) 1 bpm [−2 bpm; 3 bpm] SpO_2_ increase (before and 5 s after stimulation) 1% [−1%; 4%] 36% crying after stimulation 71% limb movements after stimulation 37% facial grimace after stimulation	
			<30 weeks of GA): Details of TS: 35% stimulation rate onset after 19 s [13 s, 32 s] episodes per infant 0 [0; 1]*	≥30 weeks of GA: Details of TS: 90% stimulation rate onset after 19 s [15 s, 24 s] episodes per infant 1 [1; 3]*
Baik-Schneditz et al.^42^	Secondary analysis of the prospective study and RCT 01/2012–12/2014	<37 + 0 weeks of GA: GA 34.9 ± 1.4 weeks *n* = 18 ≥37 + 0 weeks of GA: GA 38.9 ± 0.73 weeks *n* = 25	Analyzed time frame: first 15 min of resuscitation Preterm infants: Details of TS: 43% stimulation rate episodes per infant 1 [1; 7], overall duration 15 s [5 s; 63 s] Methods (%—of overall infants): 56% sternum 28% feet 6% back 11% several different Physiological and clinical outcomes: SpO_2_ increase of 14.2 % (30 s before and after each stimulation)* no significant change in heart rate 78% needed respiratory support	Term infants: Details of TS: 54% stimulation rate episodes per infant 1 [1; 13], overall duration 29 s [4 s; 230 s] Methods (%—of overall infants): 28% sternum 28% feet 8% back 36% several different Physiological and clinical outcomes: no significant change in SpO_2_ no significant change in heart rate 39% needed respiratory support
Pietravalle et al.^43^	Secondary analysis of the prospective study	GA 38 [37; 40] weeks *n* = 102	Analyzed time frame: depends on the infant’s transition Details of TS: 68% stimulation rate 28% infants were stimulated within first minute onset after 134 s [53 s; 251 s] overall duration 17 s [9 s; 33 s] episodes per infants 4 [2; 7], Methods (%—of episodes): 54% back rub 79% chest rub 39% abdomen rub 39% foot flick 96% truncal stimulation (chest ± back rub) Physiological and clinical outcomes: response to TS (defined as complete newborn recovery (i.e., spontaneous breathing without need for PPV) in 9% of infants (especially after rubbing the back)	
Dekker et al.^27^	Single-center RCT 09/2016–04/2017	27^0/7^–320^/7^ weeks of GA Repetitive stimulation group (defined as gently rubbing the back or the soles of the feet during 10 s, alternated with 10 s of rest): GA 29^5/7^ [28^1/7^; 30^6/7^] weeks *n* = 21 Standard stimulation group: GA 29^0/7^ [27^5/7^; 31^0/7^] weeks *n* = 23	Analyzed time frame: first 4 min of resuscitation Standard stimulation Details of TS: 96% stimulated onset after 74.5 s [±42.9 s] episodes per infant 3 [3; 6]* overall duration 59 s [24 s; 120 s] Methods (%—of episodes): 4% back rub 91% foot rub 4% both Primary outcome: minute volume after 1–4 min 51.5 ml/kg [5.3 ml|kg; 114.2 ml|kg] Physiological and clinical outcomes: average oxygen saturation 81.7 ± 8,7% * FiO_2_ at the start of transport to the NICU 0.34 [0.29; 0.44]* caffeine administration 39.1%* No statistical differences between both groups:	Repetitive stimulation Details of TS: 100% stimulated onset after 71.3 s [±34.1 s] episodes per infant 8 [7; 10]* overall duration 86 s [63 s; 105 s] Methods (%—of episodes): 1% back rub 95% foot rub 4% both Primary outcome: minute volume after 1–4 min 69.2 ml/kg [11.5 ml/kg; 153.9 ml/kg] Physiological and clinical outcomes: average oxygen saturation 87.6 ± 3.3%* FiO_2_ at the start of transport to the NICU 0.28 [0.23; 0.35]* caffeine administration 9.5%*
			MV at minutes 1–4 and 1–7; respiratory rate, tidal volume, RoR of spontaneous breaths on CPAP, percentage of tidal volumes >4 ml/kg or >8 ml/kg; pulse rate, FiO_2_, administration/duration of PPV	
van Henten et al.^44^	Prospective study	GA 34 [32; 36] weeks *n* = 40	Analyzed time frame: first 10 min of resuscitation Details of TS: 90% stimulated 48% repetitively onset after 15 s [10 s; 40 s] episodes per infant 1.5 [1; 3] overall duration 28 s [14 s; 47 s] Methods/location: 84% drying 43% rubbing 0% flicking 2.5% combination of different types 20% sternum/chest 20% back 0% feet 85% combination of different locations Physiological and clinical outcomes: no association between TS and first spontaneous breath	

Only the data of stimulated infants are depicted. Data are presented as median (IQR) or mean ± SD, asterisks (*) mark the statistically significant results.

*RCT* randomized controlled trial, *GA* gestational age, *TS* tactile stimulation, *HR* heart rate, *SpO*_*2*_ peripheral oxygen saturation, *FiO*_*2*_ fraction of inspired oxygen, *NICU* neonatal intensive care unit, *MV* minute volume, *RoR* rate of rise to maximum tidal volumes, *CPAP* continuous positive airway pressure, *PPV* positive pressure ventilation.

Outcomes were grouped together to gain a better overview and the effects of TS on vital parameters and physical response are listed.

To generate a better overview of the details on TS only data on infants that were stimulated are illustrated in the table.

## Results

### Search and inclusion results

An initial search through PubMed resulted in 271 references of potential interest. After reviewing titles and abstracts, 260 references were excluded. Full-text copies were retrieved for the resulting 11 publications. Three more papers not referring especially to delivery room management and two animal studies were excluded afterward.

Therefore, six studies were deemed eligible for inclusion and were selected for data extraction and analysis.

### Methodological quality of the final six studies

Five out of the six studies were observational, retrospective analyses of TS during neonatal resuscitation,^28,29,42–44^ one study was a RCT.^27^

The methodological quality of the RCT^27^ was assessed using the RoB 2 tool. Depending on different domains the study was rated as high risk of bias for domain two (effect of assignment to intervention and effect of adhering to intervention) and as low risk of bias for domain one (randomization process), domain three (missing outcome data), domain four (measurement of the outcome) and domain five (selection of the reported result). The overall risk of bias was rated as low.

Because of their study design, all observational studies^28,29,42–44^ were determined to have a high risk of bias.

### Characteristics of the final six studies

An overview of the six studies in humans included is shown in Table 1.

The retrospective analysis by Dekker et al. analyzed TS in preterm infants born with a gestational age <32 weeks (median [interquartile range (IQR)], 29^0/7^ [27^3/7^; 30^2/7^]) They analyzed video recordings of the first 7 min of resuscitation focusing on TS and the resulting changes in heart rate, oxygen saturation (SpO_2_), and fraction of inspired oxygen (FiO_2_) while using a respiratory function monitor.^29^

Gaertner et al. retrospectively described the differences of TS in preterm infants less and above 30 weeks of gestational age (median [IQR]; 27^0/7^ [25^0/7^;28^0/7^] vs. median [IQR]; 34^5/7^ [31^0/7^;39^0/7^]) by analyzing video recordings focusing on the effect of TS on heart rate and SpO_2_ during the first 5 min of resuscitation. The authors compared various modes of TS to determine which mode is most effective.^28^

A comparison between TS in preterm (mean ± SD; 34.9 ± 1.4 weeks of gestational age) and term infants (mean ± SD; 38.9 ± 0.73 weeks of gestational age) was performed by Baik-Schneditz et al. within a secondary analysis of video recordings obtained in a prospective observational study and a RCT.^42^ TS was analyzed during the first 15 min of resuscitation focusing on its effect on heart rate and SpO_2_.

Pietravalle et al. performed a secondary analysis of a prospective observational study and looked at TS in video recordings of term infants (median [IQR]; 38 [37;40] weeks of gestational age) during resuscitation. They focused on the effect of TS on complete newborn recovery which was defined as a spontaneous breathing infant without any need for PPV.^43^

The observational analysis of the first 10 min of resuscitation conducted by van Henten et al. focused on the relation between TS and timing of the first spontaneous breath during the first 10 min of resuscitation in infants born <37 weeks of gestation (median [IQR]; 34 [32; 36]).^44^

Based on the findings of the preceding retrospective analysis by Dekker et al. in 2017, they conducted a RCT comparing preterm infants between 27–32 weeks of gestation that were repetitively stimulated (median [IQR] GA; 29^5/7^ [28^1/7^; 30^6/7^]) with those stimulated in a standard way (median [IQR] GA; 29^0/7^ [27^5/7^; 31^0/7^]) within the first 4 min of resuscitation.^27,29^ Primary outcome was the average minute volume at 1–4 min after birth.

### Characteristics of TS

#### Stimulation rate

TS was performed in 43–90% of preterm and term infants during routine neonatal resuscitation. Stimulation was less often performed in the more immature infants compared to more mature newborns (43% vs. 54% and 35% vs. 90%).^28,42^

The highest stimulation rate (SR) of the observational, retrospective studies was seen in the study of van Henten et al. in which 90% of all preterm infants got stimulated at least once.^44^

Due to the study protocol, every infant in the intervention group of the RCT was stimulated at least once.^27^ In the control group of this trial, in which TS was applied by the discretion of the caregiver, the rate of TS was 96%.^27^

#### Onset of TS

The onset of TS during neonatal resuscitation varied widely between studies, from a median time of 15s^44^ up to 134 s after birth.^43^ A huge variability also exists within studies: Dekker et al. observed that in their unit infants were stimulated after a median time (IQR) of 114 s (73 s;182 s).^29^ Likewise, Pietravalle et al. median (IQR) onset of TS 134 s (53 s; 251 s).^43^ The incidence of stimulation within the first minute of resuscitation was inconsistently mentioned but varied between 28^43^ and 58%.^28^

#### Overall duration of TS

Infants received TS over a median period of 15 s up to 86s^27,42^ although Gaertner et al. did not describe the overall duration of TS.^28^

The median duration of TS in preterm infants ranged from 15 s^42^ up to 86 s,^27^ whereas in term infants this was described to be 17 s^43^ up to 29 s.^42^

A huge variability is seen within the studies.^27,42^ The highest variability was observed in a study of term infants with an overall duration of TS between 4 and 230 s (IQR).^42^

#### Episodes of TS

Between all studies, the median number of TS episodes per infant varied between none and eight episodes.^27,28^

In preterm infants, the median number of TS episodes ranged from none to three,^27–29^ whereas in term infants, the median number of TS episodes ranged from one to four.^42,43^

In the RCT, the median number of TS episodes differed between eight in the repetitive stimulation group and three in the standard stimulation group.^27^

#### Methods of TS

Methods of TS mentioned in the studies were chest rub, back rub, foot rub, foot flick, abdominal rub, a combination of the above or other.^27–29,42–44^ Two studies included drying as a stimulation method.^28,44^

In the studies of Dekker et al. stimulation of the feet was the preferred method,^27,29^ while in the other studies stimulation of the chest and back was preferred.^28,42–44^ In 20–79%^43,44^ of the stimulated infants, at least one stimulation was performed as a chest rub. The back was stimulated in 6% to 54% of the stimulations were performed at the back^42,43^ and the foot in 0–95%.^27,44^ Pietravalle et al. also mentioned the stimulation of the abdomen in 39% of all stimulation episodes.^43^

### Effects of TS

#### Oxygen saturation/heart rate

Vital parameters such as SpO_2_ were analyzed in three of six studies.^27,28,42^ Significant changes after TS were seen in two of the three studies.^27,42^

Gärtner et al. detected a non-significant median (IQR) SpO_2_ increase of 1% (−1%; 4%) 5 s after TS in all infants^28^ whereas Baik-Schneditz et al. outlined a significant median SpO_2_ increase of 9% (62 vs. 71%, *p* < 0.01) 30 s after TS in preterm but not in term infants.^42^ SpO_2_ was also significantly higher in infants who got stimulated repetitively during the first 4 min than in those infants who received nonspecific, standard TS (88 vs. 82%, *p* = 0.007).^27^

Four of six studies analyzed heart rate but could not find a significant change in heart rate as an effect of TS.^27–29,42^

Dekker et al. observed an increase in heart rate >100 bpm and/or an increased breathing effort in 18% of all stimulated infants.^29^

Gärtner et al. observed a change in heart rate of 1 bpm (−2 bpm; 3 bpm) 30 s after TS, which was statistically and clinically insignificant.^28^ Also, Baik-Schneditz et al. and Dekker et al. did not observe a significant change in heart rate after TS during resuscitation independent of a specific subgroup or intervention.^29,42^

#### Breathing/duration of PPV/intubation rate/oxygen requirement

Spontaneous breathing without the need for PPV was defined as a full recovery during resuscitation in one study and was reached in 8% of the infants during and after stimulation procedures.^43^ In the study of van Henten et al., no association between TS and first spontaneous breath could be assessed.^44^

In the observational study of Dekker et al., the need for intubation was significantly higher in those infants who did not receive any TS compared to those who were stimulated (18 vs. 7%, *p* < 0.05).^29^ All other studies did not mention the intubation rate.

In the RCT, there was no effect of TS on minute ventilation within the first 4 and 7 min after birth.^27^

Moreover, there was no difference in the duration of PPV in the first 7 min after birth between the repetitive stimulation group and the standard stimulation group (16 s (0–118 s) vs. 35 s (13–131 s), *p* = 0.231).^27^

Less FiO_2_ was administered at 7 min in the repetitively stimulated group compared with the standard stimulated group (0.28 vs. 0.34, *p* = 0.036).^27,42^

#### Physical activities

Only one study analyzed physical activity as a physical reaction to TS such as crying, facial grimace, and limb movements.^28^ In infants in whom the response on body movements was assessed, a change in facial grimace was seen in 37%, crying in 36%, and limb movement in 71% after TS. Physical responses were seen more often when stimulation was performed by drying and rubbing the chest or back compared with flicking the feet.^28^

## Discussion

To the best of our knowledge, this is the first systematic review on TS during neonatal resuscitation.

Although TS is common practice to stimulate spontaneous breathing of especially compromised neonates, the lack of evidence is striking.

This review identified six studies, which mainly evaluated the effects of TS on spontaneous breathing and measurable parameters such as heart rate and oxygen saturation within the first minutes after birth. Only one of the studies is a RCT comparing two different strategies of TS.

Our systematic review demonstrates the wide variability of TS during neonatal resuscitation. The studies highlight the differences between centers and patient groups. However, it still remains unclear if the performance of TS should be adjusted to the gestational age of the newborn. Preterm infants receive less TS although their respiratory drive might be more compromised after birth compared to those of term neonates. TS was relatively rare during the first minute after birth, although TS is recommended as one of the early steps of neonatal resuscitation in recent guidelines.^21^ However, many other manipulations are performed during the first minutes which could have a stimulatory effect.^30,31^

Interestingly, the effect of TS as an intervention in neonatal resuscitation is understudied. Oxygenation seems to improve after TS—especially in preterm neonates—and one RCT showed that repetitive stimulation was more effective than standard stimulation.^27^ Therefore, TS might have a “dose-depending” effect and a larger covered surface area and a longer duration might activate more mechanoreceptors which may contribute to a better clinical outcome.^45^ Even though beneficial effects of TS have been demonstrated, infants are less likely to be stimulated during PPV compared to before PPV.^45^ TS during PPV may improve spontaneous breathing and mask leak and airway obstruction do not appear to be affected by TS during mask ventilation.^45^ So far TS has not been shown to have a significant effect on important clinical outcomes even though one study suggests that intubation rates are lower in stimulated infants.

### Strengths and weaknesses of the review

This systematic review provides guidance for future research with a focus on TS in the field of delivery room management.

Only one out of six studies included in this review was a RCT.^27^ All the other studies were retrospective, observational studies describing the current clinical practice in their specific units.^28,29,42–44^ All but one study have been performed in tertiary NICUs. Therefore, the generalizability of the published studies is very limited.

The methodological quality of the studies has been assessed using established tools but the risk of bias of most of the studies is high due to their study design. Only one RCT has a low risk of bias.

### Directions for future research

Our systematic review clearly highlights the lack of evidence regarding TS after birth. During the last decades, non-invasive respiratory support in the delivery room and during neonatal intensive care medicine has gained more attention and is well established. To circumvent endotracheal intubation and mechanical ventilation in the delivery room, the establishment of a stable respiratory drive is necessary. Therefore, TS might be an important intervention, but the evidence is limited. Well-designed, high-quality research like multicenter RCTs is required in the future to specify the timing and methods of TS.

We suggest considering the following aspects:

#### Patients

The patient groups need to be clearly defined and should not include term as well as preterm infants since their response to tactile stimuli might differ and their respiratory status is very different after birth.^42^ Until now, the only high-quality data from a RCT is limited to preterm newborns between 27^0/7^ and 32^0/7^ weeks gestational age.^27^ Therefore, RCTs targeting extremely preterm newborns below 27 weeks gestational age are warranted. Term newborns with birth depression are understudied as well and a patient group of high relevance due to their high number internationally. Thus, a subdivision into at least three subgroups when considering all neonates seems reasonable.

#### Intervention

This review highlights the large variability in TS in between and within academic centers (Table 1). A future clinical study needs to clearly define the location of TS, the intensity, and the criteria for beginning and ending the intervention. Video recordings of the resuscitations would help to review adherence to the study protocol. Advanced monitoring techniques such as respiratory function monitors or respiratory inductance plethysmography can give further information regarding the efficacy of breathing and respiratory support.^46^ Since TS is often performed at different locations,^44^ the most effective location and the effect of simultaneously stimulating different locations should be studied. It remains unclear, whether TS should be used in all patients, regardless of respiratory status, and should be started immediately after birth or after a period of missing respiration.

#### Control

Defining the control intervention is challenging. Since international guidelines recommend TS as part of neonatal resuscitation, it is difficult to dispense TS completely for a control group. Nevertheless, the evidence for this recommendation is low and observational studies demonstrate that TS is not regularly performed, especially in the preterm population.^29,42^ Therefore, withholding TS for extremely preterm neonates during resuscitation might be in concordance with clinical equipoise. However, several other manipulations are performed which will have a stimulatory effect. For compromised term newborns, some TS seems necessary for a control group but clear differences regarding the intensity or location are required.

#### Outcome

The outcomes need to be clearly defined and should be objective criteria. Differences in oxygenation have been observed in the RCT^27^ and are of importance for future trials. For extremely preterm neonates, the time until onset of a stable respiratory drive or the oxygenation at 5 and 10 min of life might serve as short-term outcome parameters. Others might include intubation in the delivery room, respiratory support on admission to the NICU, or the need for mechanical ventilation throughout the first days of life. Long-term outcome parameters such as bronchopulmonary dysplasia or neurodevelopmental impairment are clinically very relevant but a large number of confounders through the NICU stay to make these outcome parameters unfeasible to achieve in a clinical trial of an intervention which is limited to the first few minutes after birth.

#### Setting

Previous research has mainly been conducted at academic centers with personnel highly trained in neonatal resuscitation. Especially for term neonates, clinical research needs to be spread into birthing units with limited access to neonatal specialists. The effect size of optimal TS might be higher in those settings. Given a large number of deliveries without the attendance of neonatal specialists, this setting is of high relevance to the community.

## Conclusion

Although it is an integral part of the current resuscitation guidelines this first systematic review on TS during neonatal resuscitation showed that the TS management widely varies between, as well as within different centers and no consensus exists which stimulation method is most effective. Some evidence exists that repetitive stimulation within the first 4 min of resuscitation improves oxygenation. We therefore propose suggestions for further studies which are warranted to optimize strategies to support spontaneous breathing after birth possibly adapted to different gestational age groups.
